# Novel electronic ferroelectricity in an organic charge-order insulator investigated with terahertz-pump optical-probe spectroscopy

**DOI:** 10.1038/srep20571

**Published:** 2016-02-11

**Authors:** H. Yamakawa, T. Miyamoto, T. Morimoto, H. Yada, Y. Kinoshita, M. Sotome, N. Kida, K. Yamamoto, K. Iwano, Y. Matsumoto, S. Watanabe, Y. Shimoi, M. Suda, H. M. Yamamoto, H. Mori, H. Okamoto

**Affiliations:** 1 Department of Advanced Materials Science, The University of Tokyo, Chiba 277-8561, Japan; 2 Department of Applied Physics, Okayama University of Science, Okayama 700-0005, Japan; 3 Institute of Materials Structure Science, Graduate University for Advanced Studies, High Energy Accelerator Research Organization (KEK), Tsukuba 305-0801, Japan; 4 National Institute of Advanced Industrial Science and Technology (AIST), Tsukuba 305-8568, Japan; 5 Division of Functional Molecular Systems, Research Centre of Integrative Molecular Systems (CIMoS), Institute for Molecular Science, Okazaki 444-8585, Japan; 6 RIKEN, Wako 351-0198, Japan; 7 The Institute for Solid State Physics, The University of Tokyo, Chiba 277-8581, Japan

## Abstract

In electronic-type ferroelectrics, where dipole moments produced by the variations of electron configurations are aligned, the polarization is expected to be rapidly controlled by electric fields. Such a feature can be used for high-speed electric-switching and memory devices. Electronic-type ferroelectrics include charge degrees of freedom, so that they are sometimes conductive, complicating dielectric measurements. This makes difficult the exploration of electronic-type ferroelectrics and the understanding of their ferroelectric nature. Here, we show unambiguous evidence for electronic ferroelectricity in the charge-order (CO) phase of a prototypical ET-based molecular compound, *α*-(ET)_2_I_3_ (ET:bis(ethylenedithio)tetrathiafulvalene), using a terahertz pulse as an external electric field. Terahertz-pump second-harmonic-generation(SHG)-probe and optical-reflectivity-probe spectroscopy reveal that the ferroelectric polarization originates from intermolecular charge transfers and is inclined 27° from the horizontal CO stripe. These features are qualitatively reproduced by the density-functional-theory calculation. After sub-picosecond polarization modulation by terahertz fields, prominent oscillations appear in the reflectivity but not in the SHG-probe results, suggesting that the CO is coupled with molecular displacements, while the ferroelectricity is electronic in nature. The results presented here demonstrate that terahertz-pump optical-probe spectroscopy is a powerful tool not only for rapidly controlling polarizations, but also for clarifying the mechanisms of ferroelectricity.

In general, ferroelectric materials can be classified into two categories; displacive type and order-disorder type^1^. Recently, it has been suggested that a transition metal oxide, LuFe_2_O_4_^2^, and an organic molecular compound, tetrathiafulvalene-*p*-chloranil (TTF-CA)^3^, show a new type of ferroelectricity, in which dipole moments produced by the variations of electron configurations are aligned. They are called “electronic ferroelectricity”, which consists of the third category of ferroelectricity^4^,^5^. In electronic-type ferroelectrics, the polarization is expected to be rapidly controlled by electric fields. Such a feature can be used for high-speed electric-switching and memory devices. Electronic-type ferroelectrics include charge degrees of freedom, so that they are sometimes conductive^3^,^6^, complicating dielectric measurements. As a result, it is difficult to evaluate the polarization magnitudes and unravel their origins in electronic-type ferroelectrics.

In the present study, we focus on an organic molecular compound, *α*-(ET)_2_I_3_, a candidate of electronic-type ferroelectrics. In *α*-(ET)_2_I_3_, ET and I_3_ molecules form layer structures, as shown in Fig. 1(a). At room temperature, the nominal valence of each ET molecule is +0.5 (Fig. 1(b)), and *α*-(ET)_2_I_3_ is a quarter-filled metal^7^,^8^. This compound shows a metal-insulator transition at 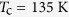
9,10,11,12,13, below which a charge-order (CO) phase consisting of ∼ + 0.7 (A and B) and ∼ + 0.3 (A′ and C) molecules with a horizontal stripe pattern is formed along the *b* axis as shown in Fig. 1(c), because of intersite Coulomb interactions^14^. In the CO phase, the crystal symmetry is *P*1 with no inversion symmetry^11^. Since the A and A′ molecules are dimerized (Fig. 1(c)), the ferroelectric polarization parallel to the *a* axis is predicted to appear^12^. However, *α*-(ET)_2_I_3_ is a good semiconductor in the CO phase, so that it is difficult to measure dielectric responses. Recently, the dielectric property including the polarization-electric-field characteristic has been studied^15^. In the study, however, the electric field was perpendicular to the ET planes. The in-plane dielectric response, which is significant to unravel the ferroelectric nature of *α*-(ET)_2_I_3_, has not been investigated because of the low resistivity. It was also revealed that second-harmonic generation (SHG) becomes active below 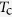 12,16. However, SHG is not an evidence of ferroelectricity because of the low symmetry (the crystal symmetry of *P*1) of this compound. Thus, the presence of an in-plane ferroelectric polarization has not been demonstrated as yet.

To overcome these difficulties, we use terahertz electric fields as external stimuli. Recent developments of femtosecond laser technology enable us to generate strong terahertz pulses^17^,^18^, which can be used for the controls of electronic states in solids^19^,^20^,^21^,^22^,^23^,^24^,^25^. Terahertz-pump SHG-probe and optical-reflectivity-probe spectroscopies on *α*-(ET)_2_I_3_, unambiguously demonstrate that the ferroelectric polarization which is inclined 27° from the horizontal CO stripe exists in the CO phase, and that this diagonal polarization originates from the collective intermolecular charge transfers. The density-functional-theory calculation qualitatively reproduced these features of the ferroelectricity. After sub-picosecond polarization modulation by terahertz fields, prominent oscillations appear in the reflectivity changes, but they are not observed in the changes of the SHG. These results suggest that the CO is stabilized by molecular displacements via the charge-phonon coupling, while the ferroelectricity is electronic in nature.

## Results

### Terahertz-pump SHG-probe measurements

**Figure 1:**
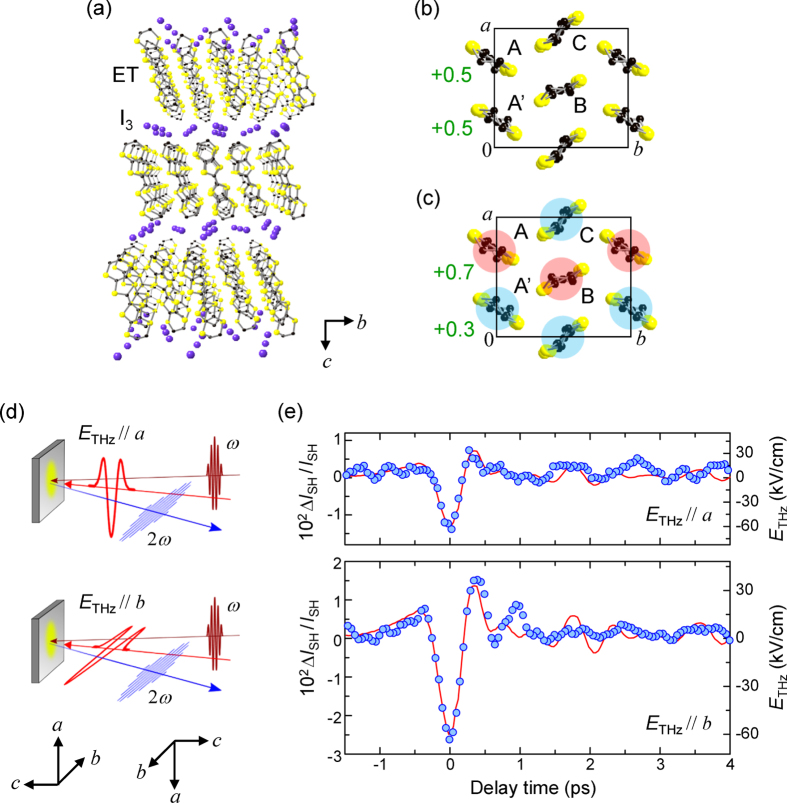
Crystal structure, CO pattern, and terahertz-pump SHG-probe measurements of *α*-(ET)_2_I_3_. (**a**) Three-dimensional map of the crystal structure. (**b,c**) Molecular arrangements and charge distributions of an ET layer in the metal phase for 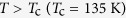 (**b**) and for 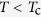 (**c**) in the right-handed coordinated system. The red and blue circles show the charge-rich (∼ +0.7) and charge-poor (∼ + 0.3) molecules, respectively. (**d**) Experimental configurations of the terahertz-pump SHG-probe experiments. The incident and SH lights are polarized parallel to *a* and *b*, respectively. Two possible directions of the crystal are shown in the lower part. (**e**) Time evolutions of terahertz-field-induced changes (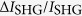) of the SH intensities 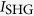 for 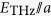 and 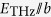 at 10 K. The red lines show the time profiles of the terahertz electric fields 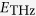.

To find evidence of ferroelectricity and clarify the origin of the ferroelectric polarization, we first performed terahertz-pump SHG-probe measurements on the *ab* plane using the reflection configurations in Fig. 1(d). Note that we could determine the directions along the three crystal axes but could not discriminate the right-handed and left-handed coordinate systems shown in the lower part of Fig. 1(d) (see Methods). The electric fields (*E*) of the incident (0.89 eV) and SH (1.78 eV) pulses were parallel to *a* and *b*, respectively, since this configuration gives the largest SHG^12^. Figure 1(e) shows the terahertz field–induced changes 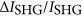 of the SH intensities 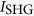, with the terahertz electric field (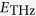) 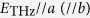 as a function of the delay time 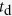 of the incident-probe pulse relative to the terahertz-pump pulse. The red solid lines show a waveform of 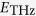, which was used as a pump pulse. The time characteristics of 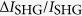 are in good agreement with the normalized terahertz waveforms, and no delayed responses are observed.

Lattice dynamics in organic molecular compounds occur on the time scale of 1 picosecond, so that they are not responsible for the sub-picosecond changes 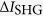 of the SHG intensities 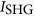. It is reasonable to consider that the 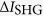 signals originate from the field-induced modulation of the ferroelectric polarization***P***. Generally, a polarization reversal by domain-wall motions in ferroelectric materials lasts much longer than 1 microsecond, which is also not the origin of the 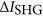. Thus, the 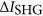 signals can be attributed to modulation in the electronic part of the ferroelectric polarization ***P***. The molecular orbital of an ET molecule was previously reported in an isolated molecule^26^, clusters^27^, *κ*-type salts^27^, and *θ*-type salts^28^. The highest occupied molecular orbitals thus reported are essentially the same with each other. The charge distribution in each molecule is almost symmetric in all cases. In addition, in *α*-(ET)_2_I_3_ the long axes of ET molecules are perpendicular to the molecular layers (the *ab* plane), so that the contributions of the intramolecular charge distributions to the observed modulations of ***P*** as well as ***P*** itself would be negligibly small. Thus, it is reasonable to consider that the modulation of ***P*** occurs through partial intermolecular CT processes, as observed in a typical electronic-type ferroelectric of an organic molecular compound, TTF-CA^24^. We performed similar measurements on several *α*-(ET)_2_I_3_ crystals, some of which showed 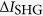 of the opposite sign. The SHG changes were observed for both 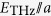 and 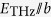, suggesting that the polarization vector ***P*** points in the diagonal direction, in contrast to the previous prediction^12^.

### Terahertz-pump optical-reflectivity-probe measurements

**Figure 2:**
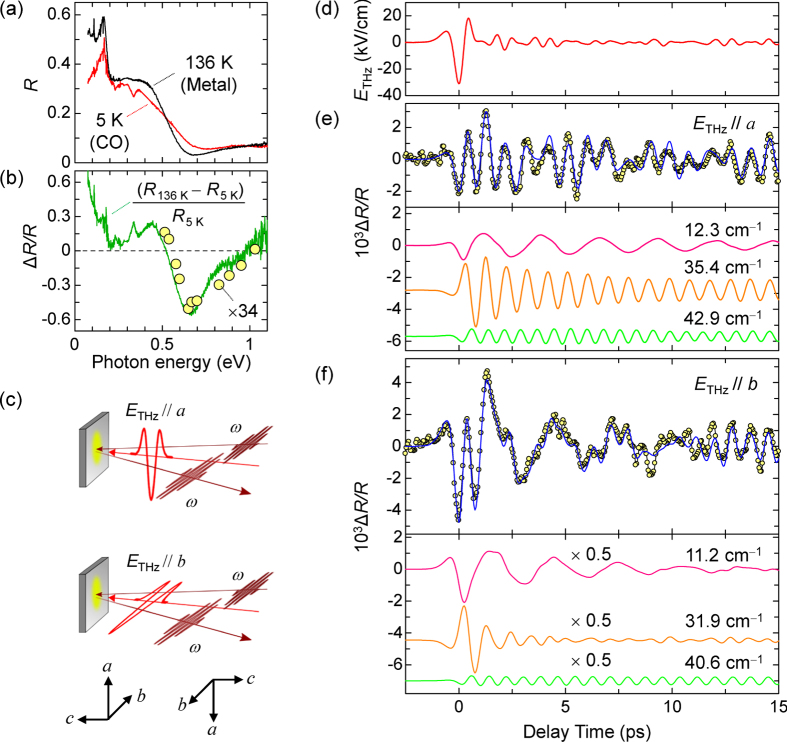
Reflectivity spectra and reflectivity changes induced by terahertz electric fields. (**a**) Reflectivity spectra at 136 K (the metal phase) and 5 K (the CO phase) for 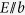. (**b**) Probe-energy dependence of terahertz-field-induced reflectivity changes 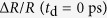 for 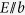 and 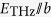 at 10 K (open circles). The maximum terahertz electric field is 100 kV/cm. The solid line shows the differential reflectivity spectrum 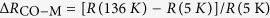. (**c**) Schematics of terahertz-pump reflection probe measurements. (**d**) A waveform of the terahertz electric field (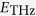). (**e**,**f**) Terahertz-field-induced reflectivity changes 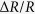 at 0.65 eV (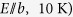 for 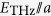 (**e**) and 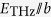 (**f**). The blue solid lines show fitting curves (see the text). The lower panels display three oscillatory components included in the fitting curves.

Next, we show the results of terahertz-pump optical-reflectivity-probe measurements, which give detailed information about the CO amplitudes related to the ferroelectric polarization***P***. Figure 2(a) shows the polarized reflectivity (*R*) spectra on the *ab* plane at 5 K (CO phase) and at 136 K (metal phase) for 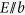. The broad band below 0.7 eV at 5 K was assigned to the CT transition between ET molecules. Its spectral shape sensitively reflects the CO amplitude and the electric conductivity^10^ (see the Supplementary Information). The solid line in Fig. 2(b) shows the differential reflectivity spectrum 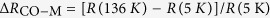 between 136 K and 5 K. 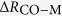 exhibits a characteristic spectrum at 0.5−1.05 eV, which corresponds to the spectral change when the CO is melted or weakened. Because ***P*** is generated by the CO, the reflectivity change should reflect changes of ***P*** as well as of the CO amplitude. Thus, in this energy region, we performed terahertz-pump reflectivity-probe experiments, which are illustrated in Fig. 2(c). The circles in Fig. 2(e,f) show the time evolution of the reflectivity changes 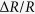 at 0.65 eV induced by the terahertz fields shown in Fig. 2(d). We discuss these results separately for the regions 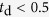 ps and 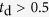 ps.

**Figure 3:**
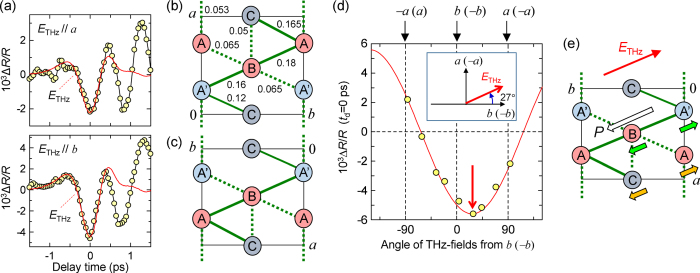
Dependence of initial reflectivity changes on the angle of the terahertz electric field. (**a**) Terahertz-field-induced reflectivity changes 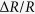 at 0.65 eV 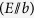 for 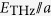 and 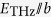 up to 1.5 ps. The red solid lines show the time profiles of the terahertz electric fields. (**b,c**) Two possible configurations of CO in the measured crystal: (**b**) the right-handed coordinated system; (**c**) the left-handed coordinated system. The red and blue circles show the charge-rich (~ +0.7) and charge-poor (~ +0.3) molecules, respectively. The numerical values indicate the transfer integrals *t* in units of eV^11^. The thick solid and dotted lines connect two molecules with large (*t* ≥ 0.1 eV) and intermediate (0.1 eV > *t* ≥ 0.05 eV) *t* values, respectively. Small *t* values (*t* < 0.05 eV) are omitted. (**d**) Terahertz-field-induced reflectivity changes 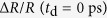 for 0.65 eV at 10 K as a function of terahertz-field angle *θ* measured from the *b* or –*b* direction. The inset shows the direction of 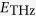 corresponding to the minimum 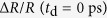. (**e**) The configuration of the measured crystal (the left-handed coordinated system) and the determined direction of ***P***. The green and yellow arrows show possible candidates for the coherent molecular oscillations with ~40 cm^−1^ and ~35 cm^−1^, respectively.

As shown in Fig. 3(a), 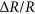 signals at 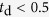 ps are reproduced well by the terahertz waveform. In fact, 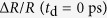 is proportional to the terahertz field at the time origin, 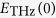 (see the Supplementary Information). The probe-energy dependence of 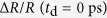 is shown by the circles in Fig. 2(b). Its spectral shape is in good agreement with 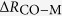, which demonstrates that the CO amplitude is weakened by terahertz fields. The ratio (∼2.1) of 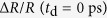 for 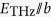 to that for 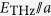 is almost the same as that (∼1.9) of 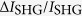 (Fig. 1e), indicating that the initial 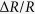 signals reflect a decrease of ***P*** and of the CO amplitude and that ***P*** is inclined from the *a* and *b* axes.

To determine the direction of ***P***, we investigated how the initial 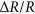 signal depends on the terahertz field direction. As mentioned above, we cannot discriminate the two crystal orientations shown in Figs 1(d) and 2(c). Therefore, we must consider two possibilities for the CO phases (Fig. 3(b,c)). Figure 3(d) shows 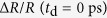 at 0.65 eV as a function of the angle 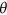 of 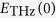 measured from *b* (Fig. 3(b)) or –*b* (Fig. 3(c)). This angle dependence is reproduced well by 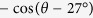, as shown by the solid line. 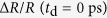 reaches its minimum at 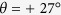 (inset of Fig. 3(d)). These results indicate that ***P*** has a diagonal direction with an angle of +27° or −153° measured from the *b* (−*b*) axis. Since ***P*** is decreased by the terahertz field when 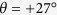, we can consider that ***P*** is directed along the −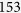 angle measured from the *b* (−*b*) axis.

As discussed above, the initial polarization modulation is attributable to the partial intermolecular CTs. It is therefore reasonable to consider that the ferroelectric polarization itself is caused by the collective CTs induced when the metal-to-CO transition occurs, similar to TTF-CA^29^,^30^. In this case, the collective CTs responsible for the ferroelectric polarization would occur between two strongly interacting neighbouring molecules. In Fig. 3(b), we show the magnitudes of the transfer integrals *t* in units of eV^11^. *t* is relatively large along the diagonal directions indicated by the solid lines connecting the A−C−A′ and A′−B−A molecules, which are inclined by +157° and +27° from the *b* axis, respectively. Assuming the specified direction of ***P*** (-153° from *b* (−*b*)), we can consider that the CT processes along the A′−B−A molecules are responsible for ***P*** because they create positive polarizations. Thus, we conclude that our experimental configuration was as shown in Fig. 3(c,e) and ***P*** had a direction *θ* = 153° from −*b* (or equivalently *θ* = +27° from *b*), as shown in Fig. 3(e).

Next, we discuss the features of the 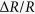 signals at 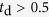 ps (Fig. 2(e,f)), in which the prominent oscillatory structures are observed. Since the oscillation frequencies are in the range 10−50 cm^−1^, they can be related to lattice modes^31^,^32^ driven by terahertz fields. To analyse the overall time evolution of 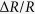, we adopt the following formula:


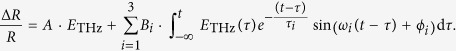


The first term represents the instantaneous response following the terahertz field. The second term is a convolution of 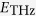 and three damped oscillators 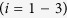 with frequency 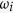, decay time 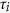, and initial phase 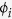. The blue lines in Fig. 2(e,f) are fitting curves, which reproduce the experimental results well. Each oscillatory component is shown in the lower panels of those figures. The oscillation frequencies (and decay times) are 12.3 cm^−1^ (11.4 ps), 35.4 cm^−1^ (5.3 ps), and 42.9 cm^−1^ (15 ps) for 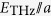, and 11.2 cm^−1^ (4.9 ps), 31.9 cm^−1^ (0.7 ps), and 40.6 cm^−1^ (56 ps) for 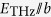. To characterize these oscillations, polarized absorption spectra were measured in the range 15–75 cm^−1^ by terahertz time-domain spectroscopy and compared with the Fourier power spectra of the time profiles (see Supplementary Information). In the absorption spectra, peaks corresponding to the coherent oscillations with ∼35 and ∼40 cm^−1^ were observed, suggesting that the coherent oscillations are related to infrared-active modes.

**Figure 4:**
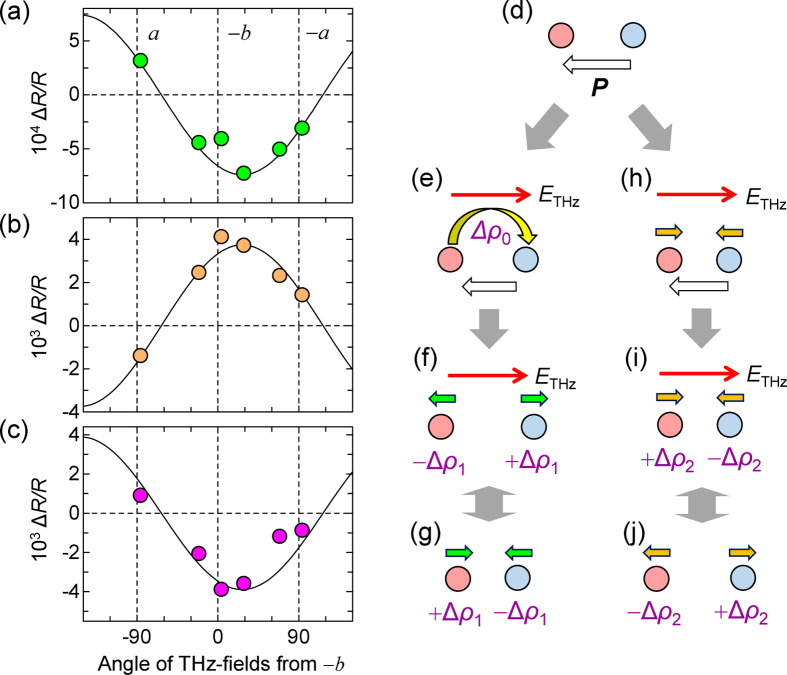
Dependence of the oscillation amplitudes on the terahertz field direction. (**a–c**) Terahertz-field-angle dependence of the amplitudes of three oscillation modes observed in 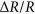 (see the text): the oscillation modes with ∼40–43 cm^−1^ (**a**), ∼32–35 cm^−1^ (**b**), and ∼11–12 cm^−1^ (**c**). (**d–j**) Simplified model to explain two kinds of coherent oscillations. The red and blue circles indicate molecules with rich and poor charges, respectively. The process d → e → f → g shows a coherent oscillation due to the charge-modulation mechanism and the process d → h → i → j a coherent oscillation directly driven by terahertz fields.

The oscillatory components exhibit the interesting feature that the initial phases of the oscillations with ∼10 and ∼40 cm^−1^ and that with ∼35 cm^−1^ are opposite to each other. To investigate this, we analysed the terahertz field–angle dependence of the initial amplitudes *B*_*i*_ in equation (1), which are shown in Fig. 4(a–c). The ∼10 and ∼40 cm^−1^ modes exhibit the same angle dependence with the terahertz field and the instantaneous charge-modulation component (Fig. 3(d)). Therefore, these modes are likely to be driven by the initial charge modulation. To explain their generation mechanism, we consider two molecules with rich (red) and poor (blue) charges, shown in Fig. 4(d). We also assume that this dimer has a finite polarization ***P*** and the terahertz field is anti-parallel to ***P***. The terahertz field induces instantaneous partial CTs, 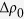 (Fig. 4(e)), which enhances the repulsive Coulomb interaction in the dimer, inducing an increase of the molecular spacing (Fig. 4(f)). Such molecular displacements will cause additional changes of the molecular ionicity, ±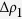, because they weaken the repulsive Coulomb interaction and destabilize the CO. Subsequently, the two molecules oscillate with a molecular ionicity modulated by 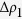 (Fig. 4(g)), which is observed as the oscillation of 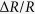.

In contrast to these oscillations, the sign of the initial amplitude of the ∼35-cm^−1^ oscillation is opposite to that of the terahertz field. Therefore, this oscillation cannot be explained by the charge-modulation mechanism. In this case, molecular displacements are considered to be driven directly by the terahertz field, as illustrated in Fig. 4(h); a terahertz field makes two molecules with rich and poor charges approach, resulting in coherent oscillation with charge modulation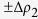 (Fig. 4(i,j)).

The time evolutions of 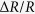 at 0.65 eV induced by the terahertz fields were measured at 50 K and 120 K as well as at 10 K (see Supplementary Information). The time characteristics of 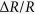 do not depend on temperature so much. This indicates that the polarization shows the same response to the terahertz fields in the CO phase below 135 K.

### Density-functional theory calculation of the ferroelectric polarization

To confirm the diagonal polarization direction in *α*-(ET)_2_I_3_ theoretically, we calculate the ferroelectric polarization based on density-functional theory (DFT). The crystalline structure at 20 K^11^ is used for the calculation. We employed a hybrid-type density functional, B3LYP, with the localized basis set 6-31 G(d) and utilized the CRYSTAL09 software^33^.

First, we demonstrate that our calculations reproduce the CO state observed at low temperatures. Table 1 summarizes the calculated molecular valencies of the four ET molecules in a unit cell and compares them with the experimental results and those of previous theoretical works. The molecular valencies in this work were estimated with the Mulliken charge analysis for spin-unpolarized and spin-polarized solutions using a *k*-mesh of 8 × 8 × 4. As shown in Table 1, the magnitude of the CO is substantially enhanced for the spin-polarized case. This result is the most comparable with the experimental one, when we consider that the calculated values are somewhat reduced owing to finite hole densities at the anions that arise from a technical reason. Note that the pure DFT calculation of Alemany *et al.* resulted in a smaller magnitude of CO compared with the experimental values^34^. In the spin-polarized solution, the total energy per unit cell was lower than that of the spin-unpolarised solution by 0.12 eV, and the spin density in each molecule had the values 0.363 (A), −0.097 (A’), −0.352 (B), and 0.086 (C). This pattern can be interpreted as an antiferromagnetic correlation between the two molecules A and B. Presumably, that correlation will lead to a spin-singlet pair, when we expand the present DFT framework to consider the correlation in detail.

Next, we calculate the electric polarization ***P*** based on the ground states determined above. Following the standard procedure of the evaluation, we change the structure from a hypothetical one with an inversion symmetry, which is parameterized as 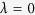, toward the actual one (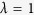), and take the difference of the polarizations as 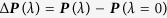. The 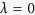 structure is generated by the symmetrization of the actual structure. Regarding the calculation of the polarization itself, we apply two methods, that are the Berry phase method^35^,^36^ and Boys method based on the Wannier functions^37^,^38^.

The *k*-mesh sampling was chosen as *n* × *n* × 4 in the calculations of 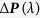. The existence of two solutions, with and without spin polarization, has also been reported for TTF-CA^29^,^30^. According to those studies, polarization calculated based on the spin-polarized solution has the same direction along the stacking axis as that determined experimentally^3^, while the spin-unpolarized one results in the opposite direction. Considering the result of TTF-CA, and the two above facts, namely, that the spin-polarized solution has the lower energy than the spin-unpolarized solution and that the former reproduces the CO pattern more satisfactorily, we report here the polarization obtained based on the spin-polarized solution.

**Figure 5:**
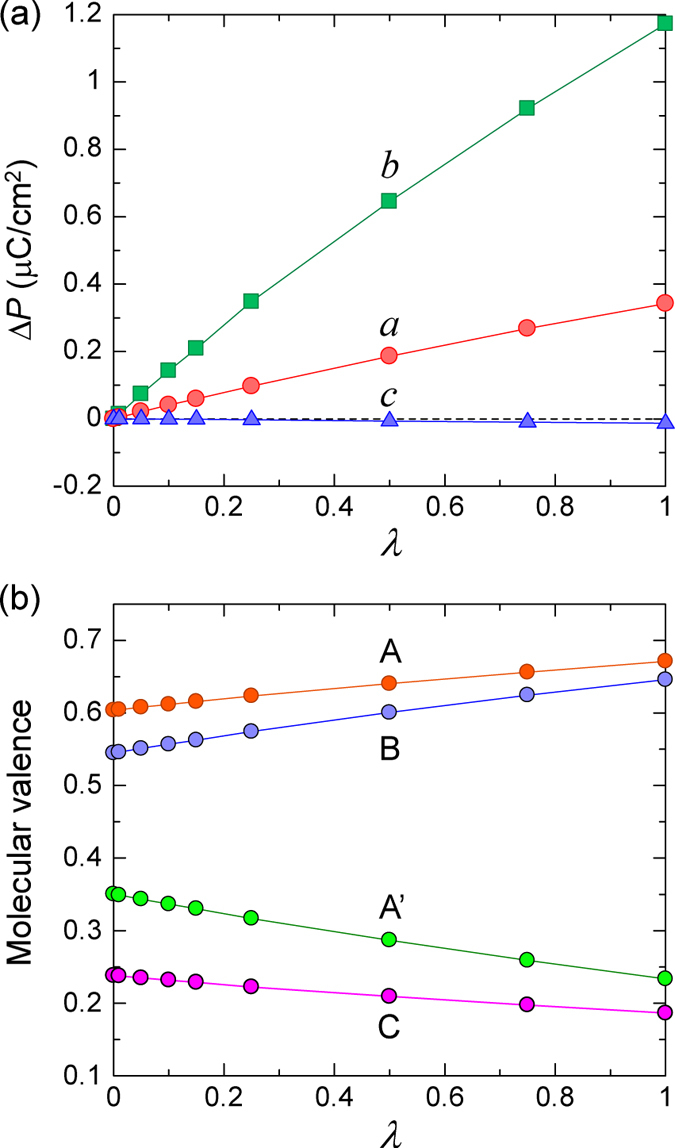
Electric polarizations and molecular valencies calculated as a function of λ. (**a**) The polarizations calculated for the structures parameterized by 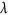. The structure 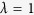 is the actual structure at 20 K, while that with 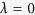 is the symmetrized structure generated from the actual structure. The k-mesh net is chosen as 8 × 8 × 4 for both the DFT calculation and the polarization evaluation. (**b**) Molecular valencies estimated for the spin-polarized solution from the summation of the atomic valencies (Mulliken charge) of the constituent atoms.

Figure 5(a) shows the components of 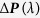 along each crystal axis for the spin-polarized solution. These were evaluated with the Berry phase method for 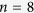. The polarization at 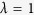 points in a direction inclined by about 16° from the *b* axis. This direction is qualitatively consistent with the experimental observation. Figure 5(b) shows the 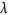 dependence of the molecular valences for the spin-polarized solution. The charge and spin ordering is mostly maintained even for the symmetrized structure, strongly suggesting its electronic origin. Such a stable CO also means the presence of the finite electric polarization at 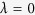. 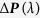 in Fig. 5(a) should be regarded as a partial polarization rather than the net one. However, we can consider that 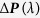 is associated with the change in the CO, since the degree of the CO changes monotonically as a function of λ, as shown in Fig. 5(b). Thus, the present theoretical result supports the idea that the direction of the polarization, which is substantially inclined in relation to the *b* axis, is dominated by hole transfer mainly from the A’ molecule to the A molecule via the B molecule.

Here, we also comment on the *k*-mesh dependence and the other calculation method. The changes in the calculated values are negligible between 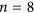 and 20, showing a good convergence. Furthermore, we confirmed that the polarizations estimated with the Boys method coincide almost perfectly with those calculated with the Berry phase method for 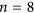. We conclude that such stable convergences come from a finite gap that survives toward 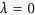; the gap energies are 0.33 eV for the up electron and 0.42 eV for the down electron for 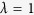 and 0.32 eV for both spins at 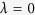.

## Discussion

In this section, we first discuss the magnitude of the polarization change, 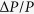, induced by terahertz electric fields, which can be evaluated from the change of the SHG intensity, 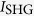. The second-order nonlinear susceptibility is proportional to 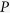, so that 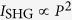 and thus 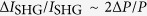. For 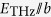, 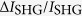 is 2.63% (Fig. 1(e)), and 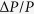 was evaluated to be 1.31% at 60 kV/cm.

The initial change of the molecular ionicity (the CO amplitude) by terahertz electric fields can be evaluated by comparing the magnitude of 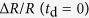 (Fig. 3(a)) with the temperature dependence of the reflectivity. When the CO amplitude *δρ* ∼±0.2 is induced at the metal-to-CO transition, the reflectivity at 0.65 eV changes by about 53%. When a terahertz field (// *b*) with 31 kV/cm is applied, 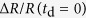 at 0.65 eV is 0.46%. Therefore, the initial change of the CO amplitude, 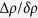, induced by the terahertz field is 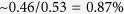 at 31 kV/cm. For 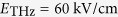, 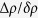 was estimated to be 1.68%, which is comparable to 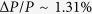, obtained from the transient SHG-probe measurement.

Next, we discuss the assignments of the oscillatory modes observed in the terahertz-field-induced reflectivity changes. The mode with ∼40 cm^−1^ is driven by the charge-modulation mechanism, so that a pair of molecules connected with large *t* is related to this mode. Considering the *t* values shown in Fig. 3(b), a possible candidate is an A′ and B pair. The ∼40-cm^−1^ mode can be related to their dimeric oscillation, as shown by the green arrows in Fig. 3(e). In contrast to the ∼40 cm^−1^ mode, the ∼35 cm^−1^ mode is driven directly by the terahertz field, so that it is related to a pair of molecules connected with small *t*. A possible candidate is an A and C pair. Thus, the ∼35 cm^−1^ mode might be attributed to the displacements of the A and C molecules, as shown by the orange arrows in Fig. 3(e). The origin of the ∼10 cm^−1^ mode is presently unclear; theoretical analyses of lattice modes based on first-principle calculations are necessary to clarify this issue. Note that coherent oscillations are hardly observed in the field-induced change of the SHG (Fig. 1(e)). This suggests that the molecular displacements responsible for the coherent oscillations stabilize the CO, but they are not coupled strongly with the polarization ***P***. This result also demonstrates that the ferroelectricity in *α*-(ET)_2_I_3_ is of the electronic type.

Finally, we discuss the effectiveness of our approach using terahertz pulses in the study of ferroelectrics. In *α*-(ET)_2_I_3_, static electric fields larger than ∼100 V/cm cannot be applied, owing to the nonlinear current flow^39^,^40^. In contrast, *α*-(ET)_2_I_3_ shows linear responses to terahertz fields at least 60 kV/cm (see Supplementary Information). Thus, the acceleration of bound carriers and additional effects, such as sample heating, never occur, owing to the short duration of the electric fields. Therefore, terahertz-pump optical-probe spectroscopy is a powerful tool not only for rapidly controlling polarizations, but also for clarifying the mechanisms of ferroelectricity.

## Methods

### Sample preparations

Single crystals of *α*-(ET)_2_I_3_ were grown using a previously reported electrochemical method^8^. The crystal orientation was determined at 294 K by X-ray diffraction measurements. In the measured crystal, we could not discriminate the *a* (*b*) and −*a* (−*b*) axes. Optical measurements were performed on the *ab* plane of the single crystals.

### Polarized reflection spectroscopy

Polarized reflection spectra of *α*-(ET)_2_I_3_ were measured using a Fourier transform infrared spectrometer equipped with an optical microscope. The samples were cooled in a conduction-type cryostat with a cooling speed of 0.3 K/min.

### Terahertz-pump SHG-probe and optical-reflectivity-probe measurements

In the terahertz-pump optical-probe measurements, a Ti:sapphire regenerative amplifier (RA) with a repetition rate of 1 kHz, a photon energy of 1.58 eV, and a pulse width of 130 fs was used as the light source. The output from the RA was divided into two beams. One was used to generate a strong terahertz pulse through optical rectification in a nonlinear optical crystal, LiNbO_3_, with a tilted-pump-pulse-front scheme^17^,^18^. The other beam from the RA was introduced into an optical parametric amplifier, from which a probe pulse (0.5−1.05 eV) was obtained. The time of a terahertz pulse was determined at the maximum of the terahertz fields. The details of the experimental setups for the terahertz-pump SHG-probe and optical-reflectivity-probe measurements and the detection method of the terahertz electric fields have been previously reported^23^,^24^.

In the terahertz-pump SHG probe measurements, we ascertained that the SH intensities 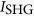 are proportional to the square of the incident-pulse intensities. In the measurements of the dependence of the reflectivity changes 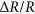 on the angle of the terahertz electric field, the electric field direction was changed by two wire-grid polarizers, which could be rotated independently. To compensate for the differences in the magnitudes of the terahertz electric fields depending on the angle, we normalized the 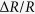 signals using the linear relation between 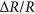 and the magnitude of the terahertz electric fields. All the experiments were performed at 10 K.

The diameters of the terahertz and optical pulses were 600 μm and 200 μm, respectively. A previous SHG-imaging study suggested that the ferroelectric domain is usually larger in size than a 200 μm square^16^. Therefore, we can detect the responses of a single domain to the terahertz fields using a probe pulse with a diameter of about 200 μm. The delay time 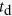 of the probe pulse relative to the pump pulse was controlled by changing the path length of the probe pulse. The time origin (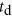 = 0 ps) was set to be the time of the maximum terahertz electric field 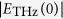.

## Additional Information

**How to cite this article**: Yamakawa, H. *et al.* Novel electronic ferroelectricity in an organic charge-order insulator investigated with terahertz-pump optical-probe spectroscopy. *Sci. Rep.* **6**, 20571; doi: 10.1038/srep20571 (2016).

## Supplementary Material

Supplementary Information

## Figures and Tables

**Table 1:** Molecular valencies of the four ET molecules in a unit cell.

	A	A′	B	C
B3LYP (spin-unpolarized)1)	0.612	0.301	0.542	0.281
B3LYP (spin-polarized)1)	0.671	0.233	0.646	0.186
PBE2)	0.638	0.438	0.577	0.359
X-ray3)	0.82	0.29	0.73	0.26

^1)^Present results.

^2)^ref. [34].

^3)^ref. [11].

## References

[b1] Lines M. E. & Glass A. M. Principles and Applications of Ferroelectrics and Related Materials (Oxford University Press, New York, 2001).

[b2] Ikeda N. *et al.* Ferroelectricity from iron valence ordering in the charge-frustrated system LuFe_2_O_4_. Nature 436, 1136–1138 (2005). 16121175 10.1038/nature04039

[b3] Kobayashi K. *et al.* Electronic ferroelectricity in a molecular crystal with large polarization directing antiparallel to ionic displacement. Phys. Rev. Lett. 108, 237601 (2012). 23003988 10.1103/PhysRevLett.108.237601

[b4] Portengen T., Östreich Th. & Sham L. J. Theory of electronic ferroelectricity. Phys. Rev. B 54, 17452–17463 (1996). 10.1103/physrevb.54.17452 9985869

[b5] Khomskii D. Classifying multiferroics: Mechanisms and effects. Physics 2, 20 (2009).

[b6] Monceau P. & Nad F. Ya. & Brazovskii, S. Ferroelectric Mott-Hubbard phase of organic TMTTF_2_X conductors. Phys. Rev. Lett. 86, 4080–4083 (2001). 11328100 10.1103/PhysRevLett.86.4080

[b7] Bender K. *et al.* (BEDT-TTF) ^+^ _2_I^−^_3_: A two-dimensional organic metal. Mol. Cryst. Liq. Cryst. 107, 45–53 (1984).

[b8] Bender K. *et al.* Synthesis, structure and physical properties of a two-dimensional organic metal, di[bis(ethylenedithiolo)tetrathiofulvalene]triiodide, (BEDT-TTF)^+^_2_I^−^_3_. Mol. Cryst. Liq. Cryst. 108, 359–371 (1984).

[b9] Takano Y., Hiraki K., Yamamoto H. M., Nakamura T. & Takabayashi T. Charge disproportionation in the organic conductor, *α*-(BEDT-TTF)_2_I_3_. J. Phys. Chem. Solids 62, 393–395 (2001).

[b10] Tajima N., Sugawara S., Tamura M., Nishio Y. & Kajita K. Electronic phases in an organic conductor *α*-(BEDT-TTF)_2_I_3_: Ultra narrow gap semiconductor, superconductor, metal, and charge-ordered insulator. J. Phys. Soc. Jpn. 75, 051010 (2006).

[b11] Kakiuchi T., Wakabayashi Y., Sawa H., Takahashi T. & Nakamura T. Charge ordering in *α*-(BEDT-TTF)_2_I_3_ by synchrotron X-ray diffraction. J. Phys. Soc. Jpn. 76, 113702 (2007).

[b12] Yamamoto K. *et al.* Strong optical nonlinearity and its ultrafast response associated with electron ferroelectricity in an organic conductor. J. Phys. Soc. Jpn. 77, 074709 (2008).

[b13] Ivek T. *et al.* Electrodynamic response of the charge ordering phase: Dielectric and optical studies of *α*-(BEDT-TTF)_2_I_3_. Phys. Rev. B 83, 165128 (2011).

[b14] Seo H. Charge ordering in organic ET compounds. J. Phys. Soc. Jpn. 69, 805–820 (2000).

[b15] Lunkenheimer P. *et al.* Ferroelectric properties of charge-ordered α-(BEDT-TTF)_2_I_3_, Phys. Rev. B 91, 245132 (2015).

[b16] Yamamoto K., Kowalska A. & Yakushi K. Direct observation of ferroelectric domains created by Wigner crystallization of electrons in *α*-[bis(ethylenedithio) tetrathiafulvalene]_2_I_3_. Appl. Phys. Lett. 96, 122901 (2010).

[b17] Hebling J., Almási G., Kozma I. Z. & Kuhl J. Velocity matching by pulse front tilting for large-area THz-pulse generation. Opt. Express 10, 1161–1166 (2002). 19451975 10.1364/oe.10.001161

[b18] Hirori H., Doi A., Blanchard F. & Tanaka K. Single-cycle terahertz pulses with amplitudes exceeding 1 MV/cm generated by optical rectification in LiNbO_3_. Appl. Phys. Lett. 98, 091106 (2011).

[b19] Kampfrath T. *et al.* Coherent terahertz control of antiferromagnetic spin waves. Nature Photonics 5, 31–34 (2011).

[b20] Liu M. *et al.* Terahertz-field-induced insulator-to-metal transition in vanadium dioxide metamaterial. Nature 487, 345–348 (2012). 22801506 10.1038/nature11231

[b21] Matsunaga R. & Shimano R. Nonequilibrium BCS state dynamics induced by intense terahertz pulses in a superconducting NbN film. Phys. Rev. Lett. 109, 187002 (2012). 23215317 10.1103/PhysRevLett.109.187002

[b22] Kampfrath T., Tanaka K. & Nelson K. A. Resonant and nonresonant control over matter and light by intense terahertz transients. Nature Photonics 7, 680–690 (2013).

[b23] Yada H., Miyamoto T. & Okamoto H. Terahertz-field-driven sub-picosecond optical switching enabled by large third-order optical nonlinearity in a one-dimensional Mott insulator. Appl. Phys. Lett. 102, 091104 (2013).

[b24] Miyamoto T., Yada H., Yamakawa H. & Okamoto H. Ultrafast modulation of polarization amplitude by terahertz fields in electronic-type organic ferroelectrics. Nature Commun. 4, 2586–2594 (2013). 24131938 10.1038/ncomms3586 PMC3826650

[b25] Kubacka T. *et al.* Large-amplitude spin dynamics driven by a THz pulse in resonance with an electromagnon. Science 343, 1333–1336 (2014). 24603154 10.1126/science.1242862

[b26] Demiralp D. & Goddard W. A. III Ab initio and semiempirical electronic structural studies on bis(ethy1enedithio)tetrathiafulvalene (BEDT-TTF or ET). J. Phys. Chem. 98, 9781–9785 (1994).

[b27] Imamura Y., Ten-no S., Yonemitsu K. & Tanimura Y. Structures and electronic phases of the bis(ethylenedithio)tetrathiafulvalene (BEDT-TTF) clusters and *κ*-(BEDT-TTF) salts: A theoretical study based on ab initio molecular orbital methods. J. Chem. Phys. 111, 5986–5994 (1999).

[b28] Kojima H. & Mori T. Dihedral angle dependence of transfer integrals in organic semiconductors with herringbone structures. Bull. Chem. Soc. Jpn. 84, 1049–1056 (2011).

[b29] Giovannetti G., Kumar S., Stroppa A., Van der Brink J. & Picozzi S. Multiferroicity in TTF-CA organic molecular crystals predicted through *ab initio* calculation. Phys. Rev. Lett. 103, 266401 (2009). 20366325 10.1103/PhysRevLett.103.266401

[b30] Ishibashi S. & Terakura K. First-principles study of spontaneous polarization in tetrathiafulvalene-p-chloranil (TTF-CA). Physica B 405, S338–S340 (2010).

[b31] Pedron D., Visentini G., Bozio R., Williams J. M. & Schlueter J. A. Phonon dynamics and superconductivity in the organic crystal *κ*-(BEDT-TTF)_2_Cu[N(CN)_2_]Br. Physica C 276, 1–8 (1997).

[b32] Iwai S. *et al.* Photoinduced melting of a stripe-type charge-order and metallic domain formation in a layered BEDT-TTF-based organic salt, Phys. Rev. Lett. 98, 097402 (2007). 17359195 10.1103/PhysRevLett.98.097402

[b33] Dovesi R. *et al.* CRYSTAL09 User’s Manual. (University of Torino, Torino, 2009).

[b34] Alemany P., Pouget J. & Canadell E. Essential role of anions in the charge ordering transition of *α*-(BEDT-TTF)_2_I_3_. Phys. Rev. B 85, 195118 (2012).

[b35] Resta R. Macroscopic polarization in crystalline dielectrics: The geometric approach. Rev. Mod. Phys. 66, 899–915 (1994).

[b36] King-smith R. D. & Vanderbilt D. Theory of polarization of crystalline solids. Phys. Rev. B 47, 1651–1654 (1993). 10.1103/physrevb.47.1651 10006189

[b37] Zicovich-Wilson C. M. & Dovesi R. *Localized functions in crystalline systems and their variational manifolds. Beyond standard Quantum Chemistry: From molecules to extended systems*, Chapter 8, (Transworld Research Network, India, 2007).

[b38] Zicovich-Wilson C. M., Dovesi R. & Saunders V. R. A general method to obtain well localized wannier functions for composite energy bands in LCAO periodic calculations. J. Chem. Phys. 115, 9708–9718 (2001).

[b39] Ivek T. *et al.* Cooperative dynamics in charge-ordered state of *α*-(BEDT-TTF)_2_I_3_. Phys. Rev. B 86, 245125 (2012).

[b40] Ito A., Nakamura Y., Nakamura A. & Kishida H. Measurement of the nonlinear conducting states of *α*-(BEDT-TTF)_2_I_3_ using electronic Raman scattering. Phys. Rev. Lett. 111, 197801 (2013). 24266489 10.1103/PhysRevLett.111.197801

