# Supplementary material for: Novel electronic ferroelectricity in an organic charge-order insulator investigated with terahertz-pump optical-probe spectroscopy
Source: Sci Rep. 2016 Feb 11;6:20571. doi: 10.1038/srep20571 (PMC4750076; doi:10.1038/srep20571)
Supplement: Supplementary Information [file srep20571-s1.pdf]

Supplementary Information

Novel electronic-type ferroelectricity in an organic charge-order insulator investigated with terahertz-pump optical-probe spectroscopy

H. Yamakawa,[1] T. Miyamoto,[1] T. Morimoto,[1] H. Yada,[1] Y. Kinoshita,[1] M. Sotome,[1] N. Kida,[1] K. Yamamoto,[2] K. Iwano,[3] Y. Matsumoto,[4] S. Watanabe,[4] Y. Shimoi,[4] M. Suda,[5] H. M. Yamamoto,[5,6] H. Mori,[7] and H. Okamoto[1]

[1]Department of Advanced Materials Science, University of Tokyo, Chiba 277-8561, Japan
[2]Department of Applied Physics, Okayama University of Science, Okayama 700-0005, Japan
[3]Institute of Materials Structure Science, Graduate University for Advanced Studies, High Energy Accelerator Research Organization (KEK), Tsukuba 305-0801, Japan
[4]National Institute of Advanced Industrial Science and Technology (AIST), Tsukuba 305-8568, Japan
[5]Division of Functional Molecular Systems, Research Centre of Integrative Molecular Systems (CIMoS), Institute for Molecular Science, Okazaki 444-8585, Japan.
[6]RIKEN, Wako 351-0198, Japan
[7]Institute for Solid State Physics, University of Tokyo, Chiba 277-8581, Japan
*Corresponding author. E-mail: okamotoh@k.u-tokyo.ac.jp

1. Comparison of optical reflectivity, electric conductivity, and CO amplitude as a function of temperature

Fig. S1 shows the temperature (T) dependence of the reflectivity, $R(T)$, of α-$(ET)_2I_3$ measured at 0.65 eV using light with an electric field (E) parallel to the b axis. This dependence is expressed as $[R(T) - R(300\ \mathrm{K})]/R(300\ \mathrm{K})$ (open circles). Below $T_c = 135$ K, $R(T)$ sharply increases, indicating the formation of the CO state. Its variation with lowering temperature is consistent with the increase of the CO amplitude evaluated by X-ray studies (triangles)[1] and also with the previously reported decrease of the electric conductivity $\sigma(T)/\sigma(300\ \mathrm{K})$ below T_c (dots)[2].

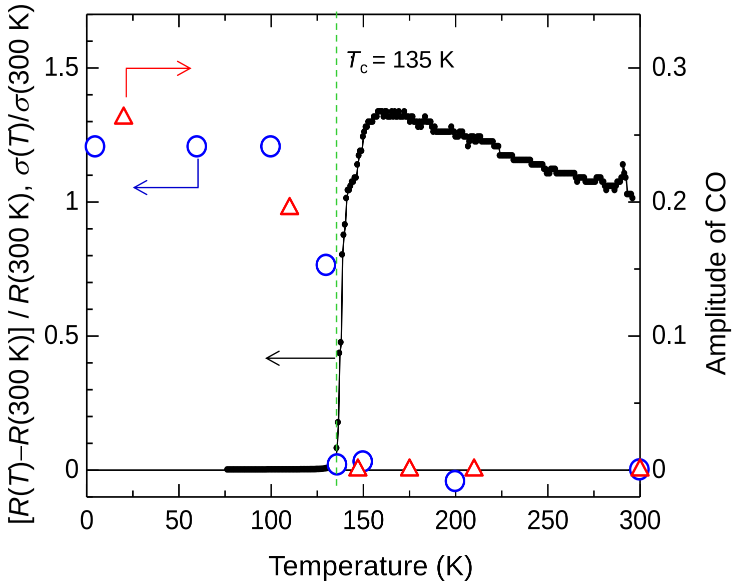

Figure S1. Comparison of optical reflectivity, electric conductivity, and CO amplitude as a function of temperature. The open circles show the temperature dependence of the reflectivity $R(T)$ at 0.65 eV. The electric field of the light (E) was parallel to b axis ($E//b$). The triangles show the temperature dependence of the CO amplitude evaluated by X-ray studies[1]. The dots show the temperature dependence of the electric conductivity $\sigma(T)$ expressed as $\sigma(T)/\sigma(300\ \mathrm{K})$[2].

2. Dependence of reflectivity changes on the terahertz electric field

We measured the reflectivity changes $\Delta R/R$ at 0.65 eV for various electric fields. Fig. S2 shows the electric field dependence of the magnitudes of $\Delta R/R$ at $t_d = 0$ ps for $E_{THz}//a$ and $E_{THz}//b$,

which were proportional to the electric field at the time origin, $E_{\mathrm{THz}}(0)$. This suggests that the observed $\Delta R/R$ signals are related to ferroelectricity.

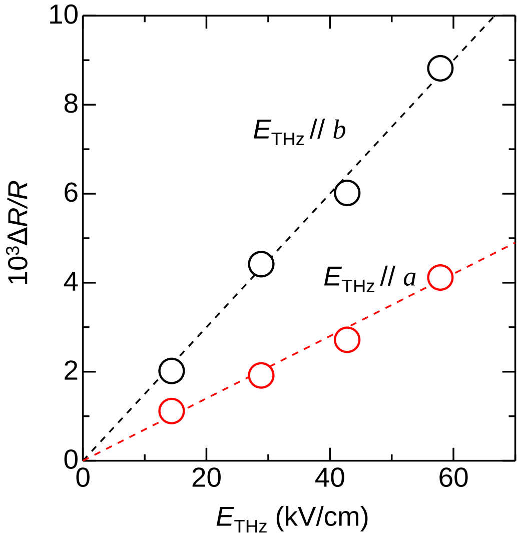

Figure S2. Terahertz-field dependence of reflectivity changes. The magnitudes of $\Delta R/R(t_d = 0\ \mathrm{ps})$ at 0.65 eV and at 10 K for $E//b$ are plotted as a function of terahertz electric field $E_{\mathrm{THz}}(0)$ for $E_{\mathrm{THz}}//a$ and $E_{\mathrm{THz}}//b$.

3. Temperature dependence of polarized absorption spectra measured with THz-TDS

To characterize the coherent oscillations observed in the time evolution of the reflectivity changes at 0.65 eV for $E//b$, polarized absorption spectra were measured in the frequency range 15–75 $\mathrm{cm^{-1}}$ using THz-TDS. The details of the experimental setup have been reported elsewhere[3].

The obtained spectra of the imaginary part ε_2 of the dielectric constant are shown in Figs. S3(a) and (b). At 137 K above T_c (=135 K), ε_2 increases with decreasing frequency, indicating metallic behaviour. For $T \leq 135$ K, the magnitudes of ε_2 are very small, indicating that the compound is insulating. Several sharp absorption peaks are observed in the ε_2 spectra for both $E//a$ and $E//b$. These peaks can be attributed to infrared-active lattice modes.

In Figs. S3(c) and (d), we show the Fourier power spectra of the coherent oscillations of the reflectivity changes for $E_{\mathrm{THz}}//a$ and $E_{\mathrm{THz}}//b$, respectively. The frequencies of the oscillatory components used in the fitting analyses shown in Figs. 2(e) and (f) are indicated by arrows. In the ε_2

spectra for $E//a$ ($E//b$), absorption peaks corresponding to the coherent oscillations with 35.4 cm^{-1} and 42.9 cm^{-1} for $E_{\mathrm{THz}}//a$, and 31.9 cm^{-1} and 40.6 cm^{-1} for $E_{\mathrm{THz}}//b$ were observed. This demonstrates that the coherent oscillations are related to infrared-active lattice modes.

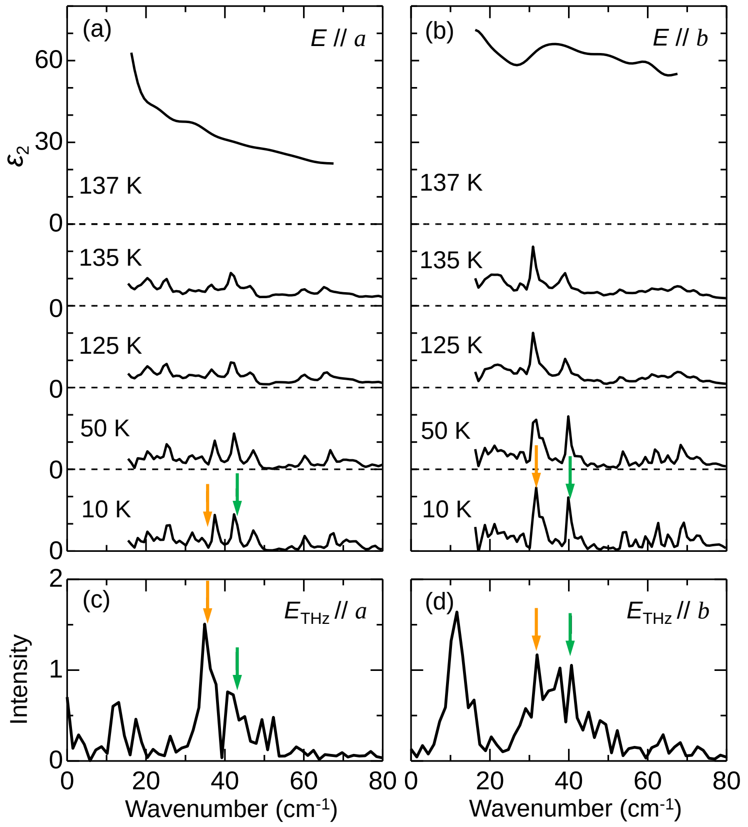

Figure S3. Polarized ε_2 spectra and Fourier power spectra of the oscillatory components of the terahertz-field-induced reflectivity changes. (a, b) ε_2 spectra for $E//a$ (a) and $E//b$ (b). (c,d) Fourier power spectra of the oscillatory components of the reflectivity changes (Figs. 2(e) and (f)) induced by terahertz fields with $E_{\mathrm{THz}}//a$ (c) and $E_{\mathrm{THz}}//b$ (d). The arrows indicate the frequencies of the oscillatory components used in the fitting analyses shown in Figs. 2(e) and (f).

The frequencies of the peaks in the ε_2 spectra are slightly different for $E//a$ and $//b$. As seen in Figs. 2(e) and (f), the frequencies and the decay times of three oscillations are also different, depending on the direction of the terahertz field. These anisotropies are qualitatively explained as follows. The magnitudes of the electric-field-induced charge transfers and their directions or, equivalently, the field-induced changes in the charge distributions should depend on the electric field direction. In this case, the frequency of each oscillation will be modified depending on the induced

charge distributions, probably through anisotropic electron-lattice interactions. As a result, the frequencies and the other oscillation parameters depend on the direction of the electric field.

4. Temperature dependence of terahertz-field-induced reflectivity changes

We performed the terahertz-pump optical-reflectivity-probe measurements with the probe energy of 0.65 eV at 50 K and 120 K as well as at 10 K. The results are shown in Fig. S4. With the increase of temperature from 10 K to 120 K, the magnitudes of the terahertz-field-induced reflectivity changes $\Delta R/R$ are almost unchanged for $E_{\mathrm{THz}}//b$ and slightly decreased for $E_{\mathrm{THz}}//a$. These temperature dependences are consistent with the fact that the temperature dependence of the CO amplitude below T_{c} (=135 K) is small as shown in Fig. S1. These result support our interpretation that the ferroelectric polarization is coupled to the CO.

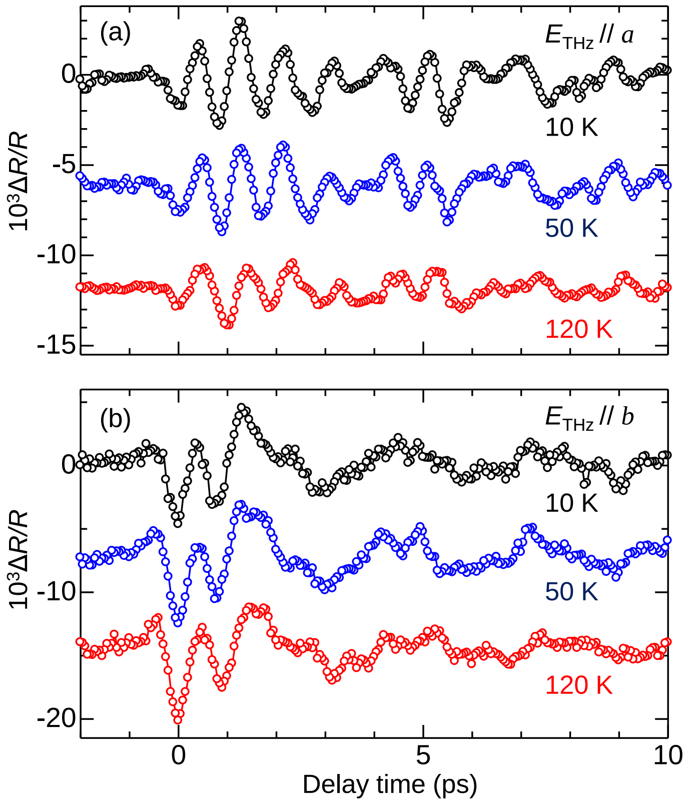

Figure S4. Temperature dependence of terahertz-field-induced reflectivity changes $\Delta R/R$. $\Delta R/R$ at 0.65 eV ($E//b$) for $E_{\mathrm{THz}}//a$ (a) and $E_{\mathrm{THz}}//b$ (b). The experimental condition is the same as that for Figs. 2(d-f) in the main text.

References

1. Kakiuchi, T., Wakabayashi, Y., Sawa, H., Takahashi, T. & Nakamura, T. Charge ordering in α-(BEDT-TTF)$_2$I$_3$ by synchrotron X-ray diffraction. *J. Phys. Soc. Jpn.* **76**, 113702 (2007).
2. Tajima, N., Sugawara, S., Tamura, M., Nishio, Y. & Kajita, K. Electronic phases in an organic conductor α-(BEDT-TTF)$_2$I$_3$: Ultra narrow gap semiconductor, superconductor, metal, and charge-ordered insulator. *J. Phys. Soc. Jpn.* **75**, 051010 (2006).
3. Takeda, R., Kida, N., Sotome, M., Matsui, Y. & Okamoto, H. Circularly polarized narrowband terahertz radiation from a eulytite oxide by a pair of femtosecond laser pulses. *Phys. Rev. A* **89**, 033832 (2014).
